# Examining long-term natural vegetation dynamics in the Aral Sea Basin applying the linear spectral mixture model

**DOI:** 10.7717/peerj.10747

**Published:** 2021-03-02

**Authors:** Yiting Su, Dongchuan Wang, Shuang Zhao, Jiancong Shi, Yanqing Shi, Dongying Wei

**Affiliations:** 1 School of Geology and Geomatics, Tianjin Chengjian University, Tianjin, China; 2 Tianjin Key Laboratory of Civil Structure Protection and Reinforcement, Tianjin, China

**Keywords:** The Aral Sea Basin, Fractional vegetation cover, The linear spectral mixture model, Google earth engine

## Abstract

**Background:**

Associated with the significant decrease in water resources, natural vegetation degradation has also led to many widespread environmental problems in the Aral Sea Basin. However, few studies have examined long-term vegetation dynamics in the Aral Sea Basin or distinguished between natural vegetation and cultivated land when calculating the fractional vegetation cover.

**Methods:**

Based on the multi-temporal Moderate Resolution Imaging Spectroradiometer, this study examined the natural vegetation coverage by introducing the Linear Spectral Mixture Model to the Google Earth Engine platform, which greatly reduces the experimental time. Further, trend line analysis, Sen trend analysis, and Mann–Kendall trend test methods were employed to explore the characteristics of natural vegetation cover change in the Aral Sea Basin from 2000 to 2018.

**Results:**

Analyses of the results suggest three major conclusions. First, the development of irrigated agriculture in the desert area is the main reason for the decrease in downstream water. Second, with the reduction of water, the natural vegetation coverage in the Aral Sea Basin showed an upward trend of 17.77% from 2000 to 2018. Finally, the main driving factor of vegetation cover changes in the Aral Sea Basin is the migration of cultivated land to the desert.

## Introduction

Vegetation dominates the terrestrial ecosystem and plays an important role in the study of global climate change. In arid areas, vegetation has essential impacts not only on precipitation and temperature but also on atmospheric circulation, water vapor transport, water balance and regional water resources (Gu et al., 2018; Zhang et al., 2019a). The Aral Sea Basin belongs to the arid area of Central Asia, with low vegetation coverage, weak anti-interference, fragile regional ecology, and obvious changes in land cover. Due to the rapid development of irrigated agriculture and climate change, the Aral Sea area has shrunk to approximately 10% of the original area, and the drastic reduction of water resources has a huge impact on the ecological environment and economic activities in the neighboring areas. Soil erosion and vegetation cover changes have seriously threatened the health and lives of residents (Crighton et al., 2010; Löw et al., 2018; Singh et al., 2018). Therefore, monitoring and evaluating vegetation cover changes in the Aral Sea Basin are important for soil erosion monitoring and global climate change and are significant for improving the local ecological environment.

Due to the sharp shrinkage and salinization of the Aral Sea, most regional studies have focused on changes in water bodies, such as measuring the inter-annual water storage changes, checking the water balance, and revealing hydrologic changes (Singh, Seitz & Schwatke, 2012; Cretaux et al., 2018; Sun & Ma, 2019). The change in water bodies in this study area has a significant impact on the formation and development of vegetation cover (Liu et al., 2020). Research has shown that the long-term rapid development of water and soil resources around the Aral Sea has led to the continuous deterioration of the ecological environment, dominated by water–the reduction of vegetation in natural oases, the intensification of desertification, and the degradation of vegetation (Indoitu et al., 2015; Shi & Wang, 2016; Xu, Wang & Zhang, 2016; Xu, Wang & Yang, 2017). Until 2019, salt storms and the decrease of aquifers destroyed 40% of the vegetation in the surrounding lands (Haag, Jones & Samimi, 2019). However, most of these studies and reports based on countries and small regions describe the current situation. It is impossible to fully understand the change in regional natural vegetation coverage without continuous observation and in-depth analysis of long-term series. In addition, few studies have separated cultivated land from natural vegetation when calculating vegetation coverage. The large-scale development of irrigated farmland in this area has a certain degree of disturbance to the water bodies and other features in the area. In this study, the dynamic driving forces of natural vegetation cover are divided into climate factors and human activity factors. Human activities determine the dynamics of cultivated land cover. It is difficult to objectively evaluate the driving factors because of the large proportion of human activities involved when discussing the dynamic reasons for vegetation cover (Jiang et al., 2017). The growth cycles of crops and vegetation differ. Cultivated land and natural vegetation can be distinguished by the phenological characteristics of sowing and harvesting (Cao et al., 2020). At present, several major questions regarding regional vegetation cover need to be answered: (1) What is the extent of vegetation coverage in this region? (2) What is the impact of the sharp decrease of water area on the natural vegetation coverage in this area? (3) What are the main driving factors affecting natural vegetation cover changes in the watershed? An understanding of these issues can contribute to the economic and social development of Central Asia. We analyzed the root causes of these changes by monitoring the long-term natural vegetation dynamics, which is of great significance to local ecological management and formulation of environmental policies.

The commonly used methods to extract vegetation coverage using remote sensing include the vegetation index method, empirical model method, and spectral mixing model (Du et al., 2013; Zanotta et al., 2014). The vegetation index method has been widely employed. However, its accuracy is relatively low, and it is not suitable for regions with complex terrain. Comparatively, the empirical model for vegetation coverage inversion has a relatively high accuracy, but high dependency on manual measurement data (Yun et al., 2010; Li et al., 2018; Gu et al., 2018). The linear spectral mixture model (LSMM) assumes that the mixed pixels are composed of several pure land cover categories (end members) (Cheng, Yuanqing & Cong, 2012). This method solves the problem of mixed pixels by obtaining the components of each end member. The nonlinear spectral mixing model is closer to the actual mixing spectrum than LSMM, but the forms of nonlinear spectral mixing models are generally complex, and many of the parameters are difficult to measure accurately. In the practical application, the simplified treatment greatly reduces the simulation of the actual spectrum. Therefore, the LSMM is selected, which is convenient, precise, and easy to implement (Wu & Murray, 2003; Weng, 2012; Deng & Wu, 2016; Zhang et al., 2019b). Because of the large basin area, it takes several days to extract vegetation coverage using LSMM through Matlab or the ENVI platform. The Google Earth engine (GEE) is a large-scale geospatial analysis platform based on cloud data that can solve the problems of slow data download, large storage, and low processing efficiency (Dong et al., 2016; Gorelick et al., 2017; Huang et al., 2017; Kumar & Mutanga, 2018). We attempt to improve the LSMM and introduce it to the GEE platform to shorten the experiment time and simplify the experiment process.

The process of trend line analysis is simple and intuitive. Regarding the nonlinear and nonstationary nature of environmental systems, Sen trend analysis and the Mann–Kendall trend test can be useful for analyzing trends in this system (Ahma et al., 2015; Nourani, Mehr & Azad, 2018). Therefore, this study uses a trend line analysis, Sen trend analysis, and the Mann–Kendall trend test to analyze the long-term natural vegetation dynamics of the study area, which lays a foundation for the subsequent discussion of the driving force analysis of natural vegetation cover.

This study involved three important steps: (1) the LSMM method was applied in GEE and aimed to distinguish between cultivated land and natural vegetation; (2) the large-scale and long-term monitoring of land cover in this area was performed; and (3) after eliminating the interference of cultivated land on the vegetation coverage, we utilized trend line analysis, Sen trend analysis and the Mann–Kendall trend test to compare and analyze the characteristics of natural vegetation cover changes in the Aral Sea Basin from 2000 to 2018. Additionally, we utilized multi-source data to explore the driving factors for change, which provides the foundation for follow-up ecological governance and agricultural development research.

## Materials and Methods

### Study areas and data

**Figure 1:**
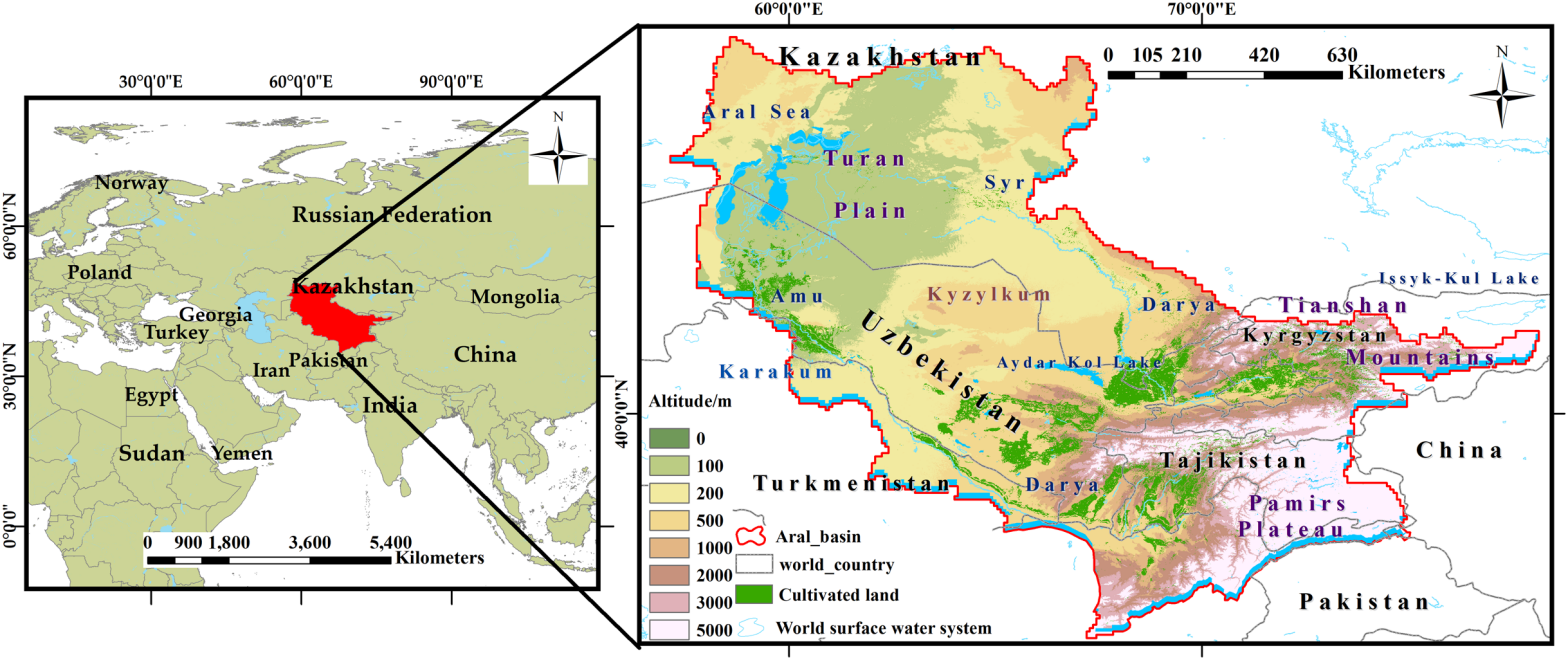
Study Area (the Aral Sea Basin).

The Aral Sea Basin belongs to the arid area of Central Asia, which is located to the west of the Qinghai–Tibet Plateau. It is composed of two major units: the Turan Plain and the mountain area. The Tianshan Mountains are in the east, the Pamirs plateau is in the southeast, the Karakum Desert is in the southwest, and the Turgai plateau is in the north, involving five Central Asian countries (Fig. 1). The Aral Sea Basin covers an area of 1.23 million km^2^. Excluding evaporation, water resources are mainly used for irrigation, forming a developed irrigation agricultural area. The main climate types are cold desert climate and temperate desert climate, with annual rainfall of less than 100 mm (Lioubimtseva, 2014; Izhitskiy et al., 2016).

**Figure 2:**
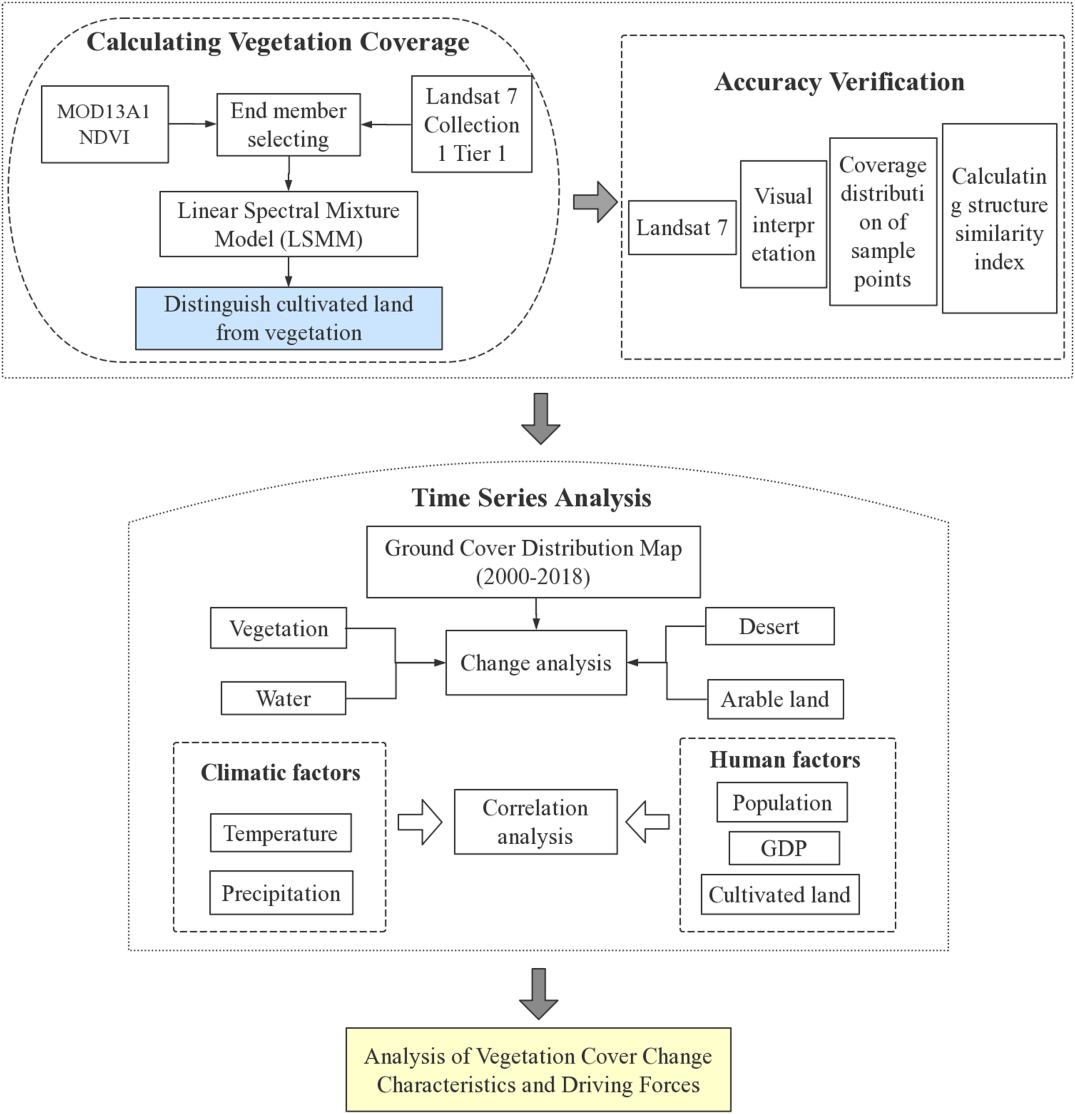
Framework applied in this study.

The data used in this study was the Moderate Resolution Imaging Spectroradiometer (MODIS) 16d synthetic NDVI data-MOD13A1, with a spatial resolution of 500 m. The RED and NIR input by MOD13A1-NDVI are the ground reflection values after atmospheric correction, and the wave amplitude is narrower, which avoids the problem of water vapor absorption in the NIR band. MOD13A1 is not only calibrated before launch, but the deviation can be constantly corrected during the process. To reduce the impact of snow, ice, and low vegetation coverage on the classification results, 266 MODIS/NDVI images were selected from April to November of each year from 2000 to 2018. Five MODIS images can cover the entire research area. The accuracy verification data was the Landsat 7 Collection 1 Tier 1. The auxiliary verification data were Google Earth high-resolution remote sensing images. In addition, temperature, precipitation, population, and GDP were selected to explore the driving forces of natural vegetation dynamics (Table 1). Framework applied in this study is shown in Fig. 2.

**Table 1:** Description of data.

Data	Data sources	Data description
MOD13A1/NDVI	https://modis.gsfc.nasa.gov/	MODIS 16 d synthetic NDVI data
Landsat 7	https://www.usgs.gov/land-resources	Spatial resolution: 30 m. Return visit period: 16 d
Google Earth Image	https://earth.google.com	Satellite image and aerial data integration, image resolution can reach 1m and 0.5 m
Meteorological data	http://wps-web1.ceda.ac.uk/submit/from?procid=Subsetter	Weather data is collected at 192 weather stations located in the Aral Sea area, including temperature and precipitation
Population, GDP data	https://data.worldbank.org/	Population and GDP data cover five countries, including Kazakhstan, Uzbekistan, Kyrgyzstan, Tajikistan and Turkmenistan
Elevation data	https://www.usgs.gov/land-resources/eros/coastal-changes-and-impacts	The data type is ASTER_GED with spatial resolution of 100 m

### Feature classification

#### Linear spectral mixture model

The mixed pixels do not completely belong to a certain kind of feature. In order to achieve higher classification accuracy, it is necessary to decompose the mixed pixels into the percentage (abundance) of a kind of feature in the pixel, that is, the decomposition of mixed pixels (Small, 2004; Deng & Wu, 2016). LSMM has obvious physical meaning, simple operation, reliable accuracy, directly related to ground coverage, and solves the problem of mixed pixels (Deng et al., 2018). The resolution of MODIS data is 0.5 km, and there is a phenomenon of pixel mixing. LSMM addresses the issue by providing valuable sub-pixel information (Zhang et al., 2019b), so we used LSMM for classification. This study is a large-scale, long-term monitoring of natural vegetation coverage, while traditional platforms require a long time to process data. Therefore, we improved the suitability of the LSMM method for MODIS data and introduced it to the GEE platform. LSMM refers to the spectral reflectance of the pixel in a certain band, which is a linear combination of the reflectance of the components that make up the pixel with the proportion of their area as the weight coefficient. This is expressed as:

(1) $$\left\{ {\matrix{ {{{R}_{i{\rm \lambda }}} = \mathop \sum \limits_{{k} = 1}^{n} {{f}_{{ki}}}{{r}_{k{\rm \lambda }}} + {{\rm \xi }_{i{\rm \lambda }}}} \cr {\mathop \sum \limits_{{k} = 1}^{m} {{f}_{{ki}}} = 1} \cr } } \right. \quad 0 \le {{f}_{{ki}}} \le 1$$

where ${{R}_{{i{\rm {\lambda}} }}}$ is the spectral reflectance of the *i*-th pixel in the λ band, ${{r}_{k{\rm \lambda }}}$ is the spectral reflectance of the ${k}$-th basic component in the ${\rm \lambda }$ band, ${{f}_{{\rm ki}}}$ is the abundance of the ${k}$-th end element in the ${i}$-th pixel; ${n}$ is the number of end elements; and ${{\rm \xi }_{i{\rm \lambda }}}$ is the residual error value, which somewhat represents the multiple reflection and transmission of light between image tuples. It has a nonlinear mixing effect. ${n}$ is the number of end elements (Wu & Murray, 2003).

#### Endmember selecting

It is important to select the end element in the LSMM. The quality of the end element directly affects the overall accuracy of the experiment. Generally, three to four end elements are selected through tests, and the inversion effect is good (Zanotta et al., 2014). Because the image will be affected by the atmosphere, terrain, and sensors, the actual spectral curve differs from the spectral curve in the ground measurement and ground object spectral library, so it is more accurate to obtain the end element from the image (Zare & Ho, 2014). There are many methods to select image endmembers. But the disadvantage of the PPI algorithm is that the selected vector has a high degree of arbitrariness; the performance of the N-FINDR algorithm is largely related to the nature of the initially selected endmember (Zhong, Zhao & Zhang, 2014; Jafarzadeh & Hasanlou, 2019); and the nature of the AMEE algorithm also depends on the relationship between the spatial characteristics of the structure element and the spectral distribution in the scene (Li et al., 2011). Generally speaking, all kinds of endmember extraction algorithms are still in the exploratory stage, and each algorithm has its own advantages and disadvantages, which needs further improvement. Here, we tended to choose the final elements with a basis to improve the accuracy of the classification results, and visual interpretation is also widely used to verify the accuracy of machine classification (Yu et al., 2017), so visual interpretation was selected for endmember selection (Feng et al., 2019; Jiang, Van der Werff & Van der Meer, 2020). The specific experimental steps were (1) MOD13A1 was overlapped with Landsat 7 and Google Earth images; (2) the pure ground object pixel of Landsat 7 and Google Earth images were interpreted and recognized visually; and (3) the pixel values in MOD13A1 at the same position as the pure ground object pixel were taken as the end element values.

**Figure 3:**
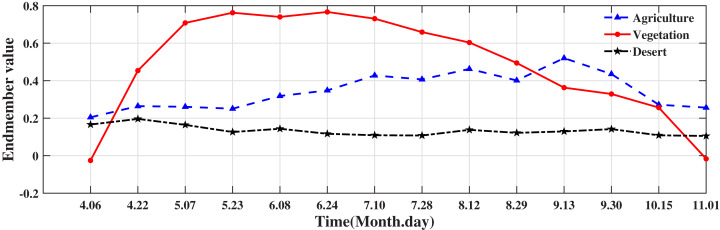
Endmember value. Endmember categories: agricultural land, desert, and natural vegetation. Choosing the end element value of 16 days from April to November can better reflect the regional annual phenological changes. The figure shows the end element value of the ground feature in 2018.

According to the distribution of ground features in the study area and the principle of selecting end elements (Micklin, 2016), four types of end elements were selected: cultivated land, vegetation, desert, and water body. The experiment selected 1,064 end elements from April to November in each year from 2000 to 2018. The water body of the MOD13A1 product was Nodata. Figure 3 shows the other end member values in 2018. The endmember values of each month in other years were selected using the same method.

### Accuracy verification

Structural similarity index model (SSIM) is a common image quality evaluation method used as an index to measure the similarity between two images (Brunet, Vrscay & Zhou, 2012). This method calculates the error between the corresponding pixels of the two images and divides the image evaluation into three parts: brightness comparison, contrast comparison and structural similarity. Therefore, this method considers not only the coverage area but also the location allocation (Li & Bovik, 2010; Ma et al., 2020). This method fully considers the correlation between human visual characteristics and images, which is an effective and correct image quality evaluation standard in line with the characteristics of the human visual system, and the subjective evaluation results are highly consistent. Attempt to imitate the metric of the human visual system-Structural Similarity Index with a local method, which was used as a comparative statistic for exploratory maps (Robertson et al., 2014). In recent years, it has been widely used (Okarma, Jarosław & Mateusz, 2016). The formula for calculating structural similarity is as follows:

(2) $${\rm SSIM} = \displaystyle{{\left( {2{{\rm \mu }_1}{{\rm \mu }_2} + {{\rm C}_1}} \right)\left( {{{\rm \sigma }_{12}} + {{\rm C}_2}} \right)} \over {\left( {{\rm \mu }_1^2 + {\rm \mu }_2^2 + {{\rm C}_1}} \right)\left( {{{\rm \sigma }_1} + {{\rm \sigma }_2} + {{\rm C}_2}} \right)}}$$

where ${{\rm \mu }_1}$ is the average value of the spectral pixel decomposition model fraction; ${{\rm \mu }_2}$ is the average value of the visual interpretation fraction of Landsat 7; ${{\rm \sigma }_1}$ and ${{\rm \sigma }_2}$ are the variance between the modeled fraction and the visual interpretation fraction, respectively; ${{\rm \sigma }_{12}}$ is the covariance; and ${{\rm C}_1}$ and ${{\rm C}_2}$ are constants. The closer the SSIM value is to 1, the higher the similarity between the modeled fraction and the visual interpretation fraction (Dosselmann & Yang, 2011; Wang et al., 2014).

### Time series analysis

#### Trend line analysis

Regression analysis is a mathematical model used to study the relationship between multiple variables and is widely employed in long-term studies and analysis of land cover change (Zhu et al., 2019; Parvin, 2019). The mathematical model implemented in this study was:

(3) $${y} = {kx} + {a} + \partial$$

where *a* and *k* are unknown constants, $\partial$ is the error, and *x* is the time independent variable. *y* is the dependent variable for ground object cover areas, *k* can be calculated by the *x*, *y* observations:

(4) $${k} = \displaystyle{{\mathop \sum \nolimits_{{i} = 1}^{n} \left( {{{x}_{i}} - \displaystyle{1 \over {n}}\mathop \sum \nolimits_{{i} = 1}^{n} {{x}_{i}}} \right)\left( {{{y}_{i}} - \displaystyle{1 \over {n}}\mathop \sum \nolimits_{{i} = 1}^{n} {{y}_{i}}} \right)} \over {\mathop \sum \nolimits_{{i} = 1}^{n} {{\left( {{{x}_{i}} - \bar {\rm x}} \right)}^2}}}$$

where *n* is the year.

The slope of the equation *k* > 0 indicates that the ground cover increases with the year. The fitting accuracy is checked using the decision coefficient *R*^2^. The closer *x* is to 1, the higher the goodness of fit.

#### Sen trend analysis and Mann–Kendall trend test

Sen trend analysis can calculate the trend degree, and the Mann–Kendall trend test can test the significance of the change trend (Drápela & Drápelová, 2011). The combination of the two methods has formed a new long time series trend analysis method, which can reduce the impact of data noise (Bi et al., 2020). The Sen trend analysis combined with the Mann–Kendall test can be utilized to determine the variation trend of long-term series data (Jiang et al., 2015). The calculation formula is:

(5) $${\rm \beta } = {\rm Median}\left( {\displaystyle{{{{x}_{j}} - {{x}_{i}}} \over {{j} - {i}}}} \right),\quad {j}\;\gt i,i = 1,2,3 \ldots ,N$$

Among, ${{x}_{j}}$ and ${{x}_{i}}$ are elements of the time series of the trend to be analyzed. When β > 0, it indicates an upward trend; when β < 0, it indicates a downward trend.

Mann–Kendall trend test does not require the test data to follow certain distribution rules, and can avoid the influence of a few outliers. The test statistic S is calculated as follows:

(6) $${S} = \sum\limits_{{K} = 1}^{{n} - 1} {\sum\limits_{{j} = {k} + 1}^{n} {{\rm Sgn}} }\,({{X}_{J}} - {{X}_{K}})$$

Inside,

(7) $${\rm Sgn}\,({{X}_{J}} - {{X}_{K}}) = \left\{ {\matrix{ { + 1\; {{X}_{J}} - {{X}_{K}} \gt 0} \cr {0\; \; {{X}_{J}} - {{X}_{K}} = 0} \cr { - 1\; {{X}_{J}} - {{X}_{K}} < 0} \cr } } \right.$$

*S* is a normal distribution and the mean value of 0, variance ${\rm Var}\left( {S} \right) = \textstyle{{{n}\left( {{n} - 1} \right)\left( {2{n} + 5} \right)} \over {18}}$, When *n* > 10, the standard normal variable is calculated as follows:

(8) $${Z} = \left\{ {\matrix{ {\displaystyle{{{S} - 1} \over {\sqrt {{{V}_{{ar(S)}}}} }}\quad S > 0} \cr {\quad 0\quad S = 0} \cr {\displaystyle{{{S} + 1} \over {\sqrt {{{V}_{{ar}\left( {S} \right)}}} }}\quad S < 0} \cr } } \right.$$

In this study, the confidence level α = 0.01 or α = 0.05, *Z* > 0 was the rising trend; *Z* < 0 is a downward trend, and when |*Z*| ≥ 1.96 and |*Z*| ≥ 2.576, the reliability was 95% and 99%, respectively (Gocic & Trajkovic, 2013).

#### Principal component analysis

Principal Component Analysis (PCA) is a statistical analysis method that converts multiple variables into unrelated comprehensive variables (Zhang et al., 2020). The basic principle of PCA is to ensure the minimum loss of information, reduce the dimensionality of the original data, and improve research efficiency while ensuring research accuracy (Bunnell et al., 2020). PCA as a descriptive tool needs no distributional assumptions and, as such, is very much an adaptive exploratory method which can be used on numerical data of various types (Jolliffe & Cadima, 2016). Its mathematical model is as follows:

(9) $${X} = \left( {{{X}_{{ij}}}} \right){A*B},{i} = 1,2, \cdots ,{B}$$

*A* is number of study areas, original sample matrix *X* of *B* selection indicators.

Calculate the correlation coefficient matrix between each indicator ${{R}_{{b*b}}}$, eigenvalues and normal eigenvectors ${{e}_{j}}$, this gives the principal component ${{T}_{i}}$, as follows: ${{T}_{i}} = {{X}_{{ej}}}$.

The contribution rate formula is as follows: ${a} = \mathop \sum \limits_{i}^{q} {{a}_{j}}$

When the j-th principal component variance contribution rate is 85–95%, the original B index information can be reflected. At the same time, the comprehensive score of ecological security in the study area can be obtained, as follows: ${W} = {a}{{X}_1} + {b}{{X}_2} + \cdots + {x}{{X}_{x}}$, where *X* is eigenvectors; *a*, *b*, … , *x* are standardized data of the original data (Licciardi et al., 2012).

## Results

### Spatio-temporal variation characteristics of natural vegetation cover

**Figure 4:**
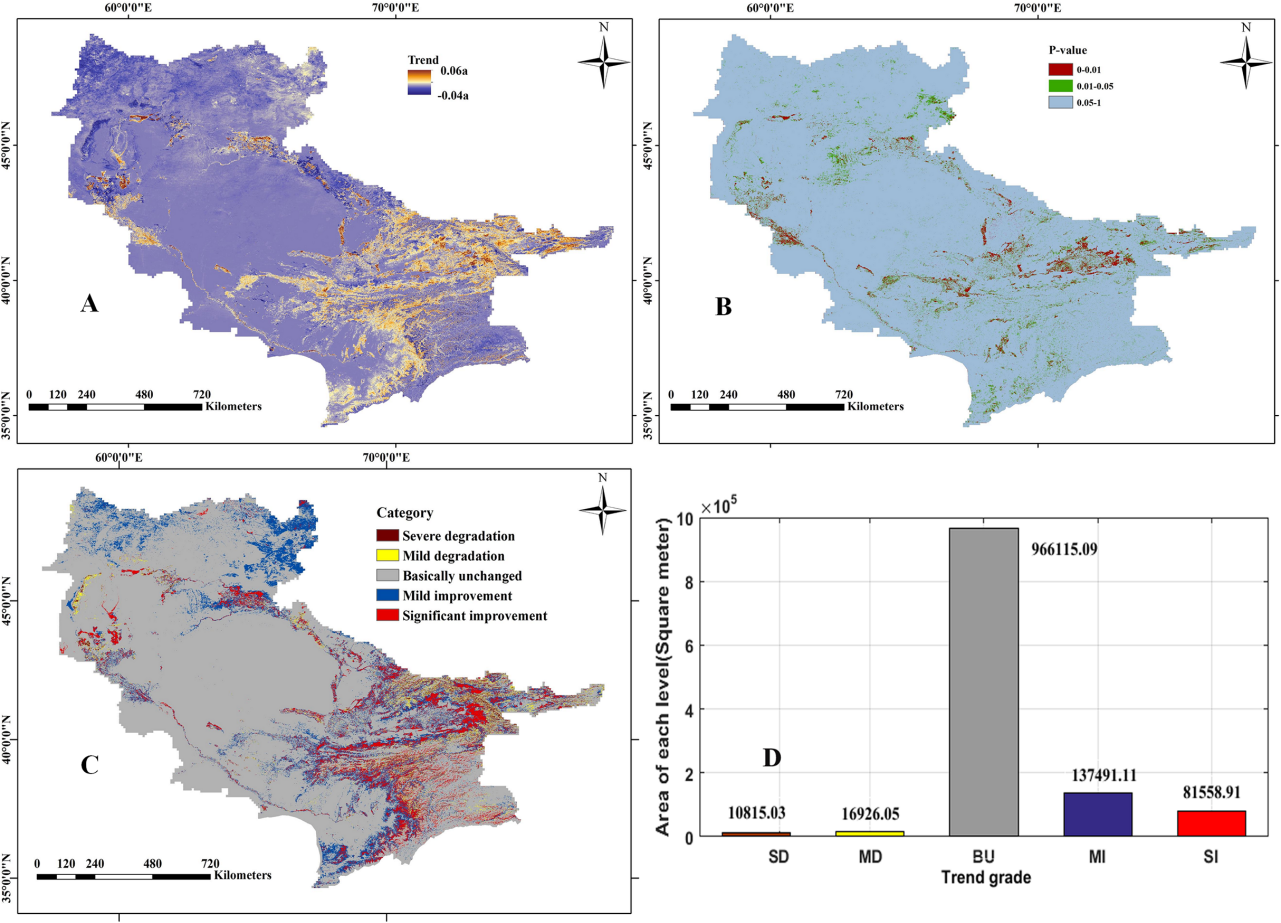
Change trend of natural vegetation coverage from 2000 to 2018. (A) Annual change trends in natural vegetation coverage; (B) significance (*p*-value) of the change trends in natural vegetation coverage; (C) hierarchical map of the change trend (the trend classified into five types: SD, severe degradation; MD, mild degradation; BU, basically unchanged; MI, mild improvement; SI, significantly improvement); (D) statistical results of cover area for different vegetation types.

Based on the GEE platform, the abundance map of the ground objects calculated by LSMM was extracted. The vegetation coverage in the Aral Sea Basin was generally lower. According to statistics, in the past nineteen years, the average area of vegetation cover in the Aral Sea Basin was 77,200 km^2^, accounting for 6.26% of the total drainage area, and the coverage was generally poor (Figs. 4A and 4B).

**Figure 5:**
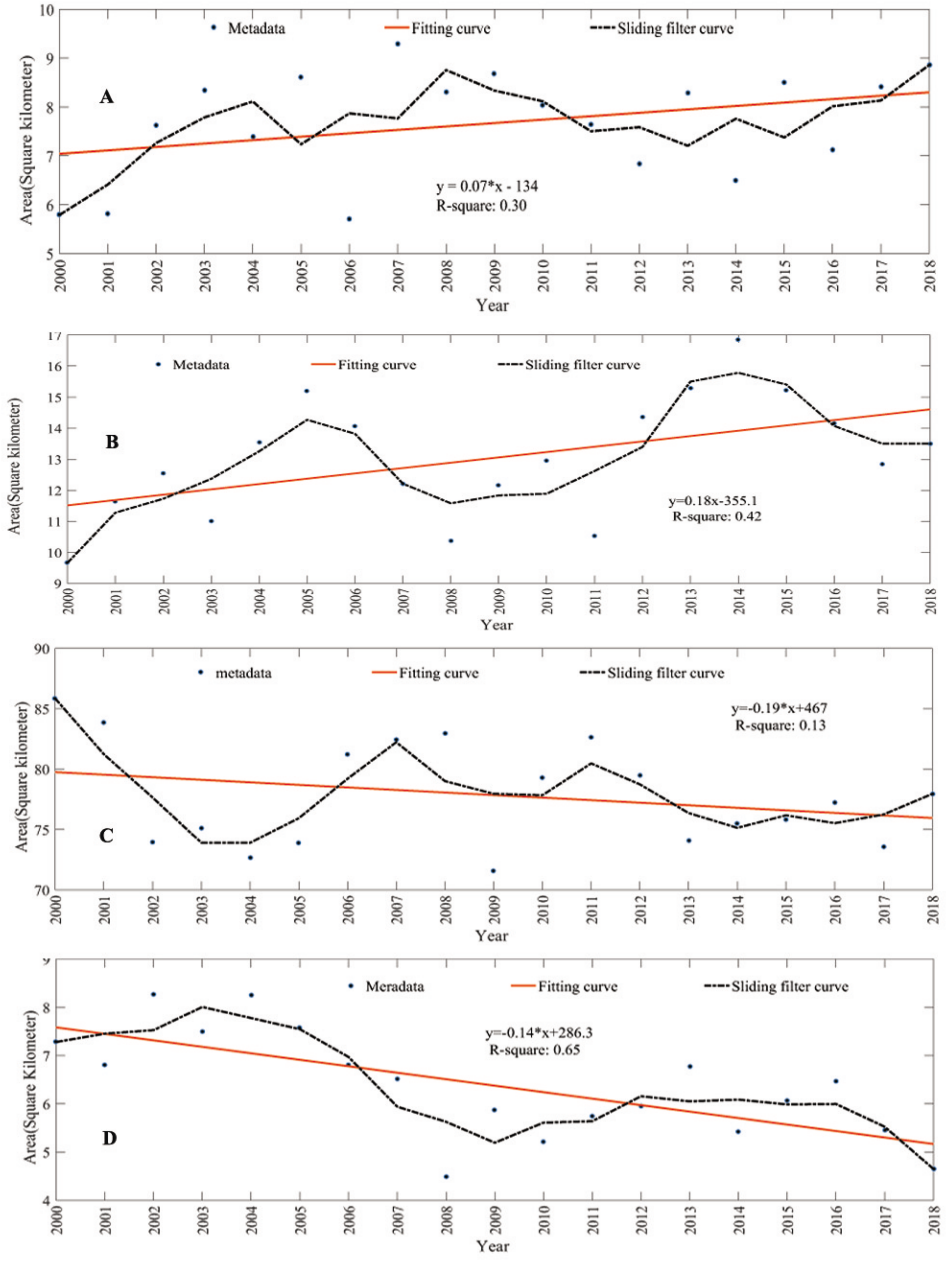
Change of land cover area in the Aral Sea Basin. (A) Natural vegetation; (B) cultivated land; (C) desert; (D) water.

To reduce the outliers and ensure the accuracy of the data, the mean sliding filter model was used. A sliding filter model with an interval of three years was used to process the annual average vegetation coverage data of the Aral Sea Basin in the past nineteen years, and the corresponding linear equations were fitted using the least squares method. The fitting results are shown in Fig. 5A. The slope of the regression equation was *k* > 0. From the results of the Sen trend analysis and Mann–Kendall trend test (β = 579.14 > 0, *Z* > 0), the vegetation coverage had an upward trend in the past twenty years. The average vegetation coverage increased from 4.70% in 2000 to 7.19% in 2018, an increase of 2.49%. The growth rate from 2000 to 2003 was large, with an increase of 25,400 km^2^. During the same period, the water bodies also increased significantly, providing sufficient water sources for vegetation growth. In 2006 and 2009–2012, there was a decreasing trend. The change was the most significant in 2006. The area of the reduction was 18,500 km^2^. During the same period, the desert area showed a rapid expansion trend. The area of vegetation coverage increased by 12,900 km^2^ from 2000 to 2018, and the area of decreased by 12,300 km^2^. The coverage of natural vegetation generally increased.

Through superposition analysis and difference calculation of the generated abundance map, the percentage change of vegetation in each pixel of the Aral Sea Basin in the last nineteen years was obtained. According to the change range, the vegetation change in the Aral Sea Basin was divided into five categories: severe degradation, mild degradation, basically unchanged, mild improvement, and significant improvement by using the standard deviation classification method; this can be seen in Figs. 4C and 4D. In the past nineteen years, the vegetation coverage of the Aral Sea Basin remained unchanged, and the area where the vegetation coverage has significantly improved is larger than the degraded area. The changes and spatial distribution of each category are shown in Table 2. The overall fluctuation of vegetation coverage in the Aral Sea Basin is small, showing low central and high east and west features. The high fluctuation range (severely degraded and significantly improved areas) is small, accounting for 7.49% of the basin, mainly concentrated in the mountains and rivers. The improvement area is more obvious (red part), and the improvement area greatly exceeds the degraded area (improved 17.77%, degradation only 2.24%).

**Table 2:** Classification criteria and statistical results of natural vegetation trend.

Types	Range	Pixels	Area/km^2^	Percentage/%	Bistribution area
SD	<−0.1790	54,062	10,815.03	0.87	North of Tianshan Mountain
MD	−0.1790 to −0.1611	434,127	16,926.05	1.37	Eastern Tianshan and western Amu Darya delta
BU	−0.1611 to 0.6533	4,363,687	966,115.09	79.98	Turan Plain and desert area
MI	0.6533–0.2282	758,782	137,491.11	11.15	The northwest of the basin and the north of the Pamirs
SI	0.2282–1.0000	159,934	81,558.91	6.62	Eastern mountain and delta coast

**Note:**

The range is the pixel vegetation abundance value.

### Analysis of land coverage changes

#### Spatio–temporal variation characteristics of ground cover

**Figure 6:**
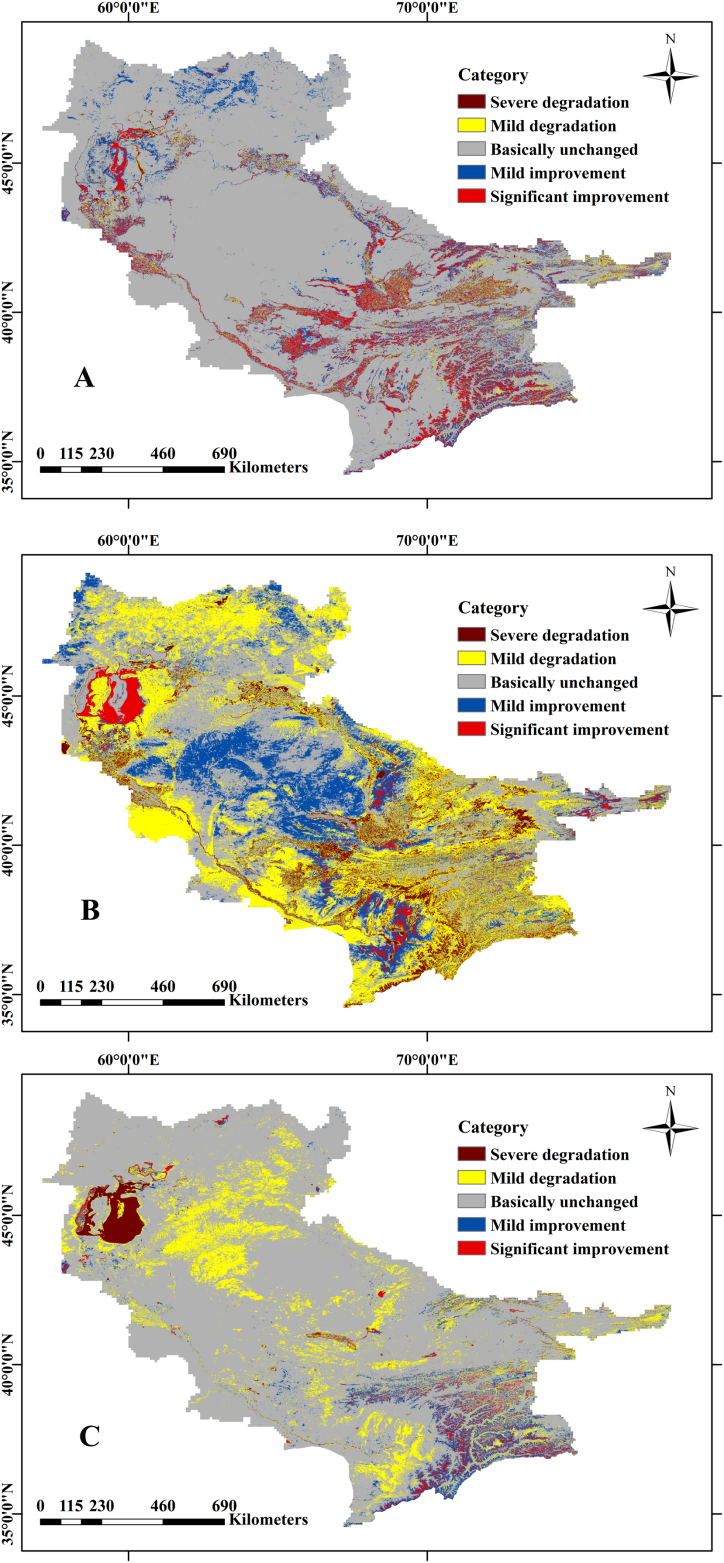
Hierarchical map of the change trend of the land coverage. (A) Cultivated land; (B) desert; (C) water.

In order to better explore the driving forces affecting natural vegetation coverage, this study quantitatively analyzes the dynamic changes in the abundance maps of other features of the Aral Sea Basin (Table 3). The average area of cultivated land in the Aral Sea Basin was 136,600 km^2^, accounting for 10.54% of the total basin area. The area of cultivated land increased from 96,600 km^2^ in 2000 to 135,000 in 2018, an increase of 3.10%. The growth rate was large in 2000–2005 and 2011–2014, and the total increase in area was 166,000 km^2^, mainly distributed on both sides of the Amu Darya, upstream of the Syr Darya, and the Zeravshan River. The reduction was large in 2005–2008 and 2014–2017, with an area reduction of 127,600 km^2^, mainly distributed in Kokand and northern Tashkent. Overall, the distribution decreased inside the city and increased around the city periphery and the river (Figs. 5B and 6A).

**Table 3:** Statistical results of land cover types in the Aral Sea Basin (unit: %).

Land cover types	SD	MD	BU	MI	SI
Desert	8.75	33.14	39.07	16.05	2.98
Cultivated	1.48	9.59	73.02	8.74	7.17
Water	1.95	12.85	78.30	4.82	2.09
Vegetation	0.87	1.37	79.98	11.15	6.62

The average desert area of the Aral Sea Basin is 778,300 km^2^, accounting for 62.85% of the basin area, nearly 3/5. From 2000 to 2018, the average annual desert area decreased from 858,500 km^2^ to 778,300 km^2^, a decrease of 6.47%. The reduced area is striped along the Aral Sea basin boundary. From 2005–2008 and 2009–2011 there was an increasing trend, with a total growth area of 300,600 km^2^. The more obvious increase was observed around the South Aral Sea. As the lake subsided, new deserts formed around the edge. The distribution of deserts decreased around the edge and increased in the center (Figs. 5C and 6B).

The average area of the water body in the Aral Sea Basin was 63,800 km^2^, accounting for 5.15% of the entire basin area, and its coverage was low. It decreased from 72,500 km^2^ in 2000 to 46,400 km^2^ in 2018, a decrease of 2.11%. The reduction was largely in 2004–2008, with a total area reduction of 88,400 km^2^. The primary reason is that the amount of water in the South Aral Sea has decreased dramatically. The increased water areas are mainly distributed in the North Aral Sea and the Pamirs. The reason for the reversal of the South Aral Sea and the North Aral Sea is that Kazakhstan built a dam in 2005 to introduce the Syr Darya from the South Aral Sea to the North Aral Sea. Over the past nineteen years, water bodies reduced by a total area of 22,300 km^2^ more than the increase in area, and the water body is decreasing (Figs. 5D and 6C).

#### Correlation analysis of desert and cultivated land cover change

**Figure 7:**
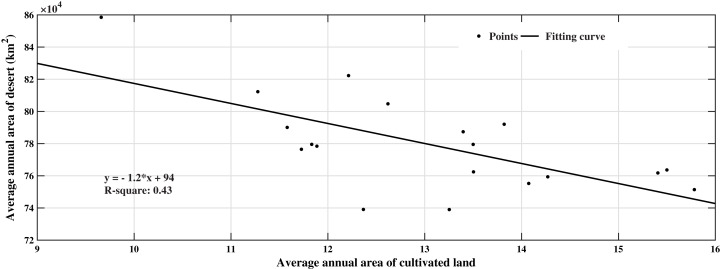
Correlation analysis between cultivated land area and desert area.

By analyzing the inter-annual spatial distribution characteristics of the cover area of cultivated land and deserts, we obtained a slope of *k* < 0 and a determination coefficient of 0.44 (Fig. 7). This shows that the change in cultivated land coverage has a significant relationship with the desert area. Areas with significantly increased cultivated land coverage showed an expanding trend from the original farmland to the surrounding areas (the red part in Fig. 6A). The desert shows a degrading trend (black part in Fig. 6B). The spatial distribution of desert degradation areas and farmland growth areas is similar, mainly distributed along the banks of the Amu Darya, deltas, and around cities. In desert areas, the temperature is relatively high, and the surface water evaporates quickly which limits the growth of crops. The development of irrigated farmland in this area requires a large amount of river water to be directed to the cultivated land, and the soil in Central Asia has a high salt content. In order to reduce the salinity, cultivated land must be ‘washed’ regularly. According to statistics, more than 1 billion km^3^ of water is used to clean farmland every year. A large amount of surface runoff is used for irrigation, resulting in a decrease in the amount of water in the lower Amu Darya and Syr Darya Basin, which exacerbated the shrinkage of the Aral Sea (Fig. 6C).

### Analysis of driving forces of natural vegetation cover

#### Impact of climate variability on natural vegetation cover

**Figure 8:**
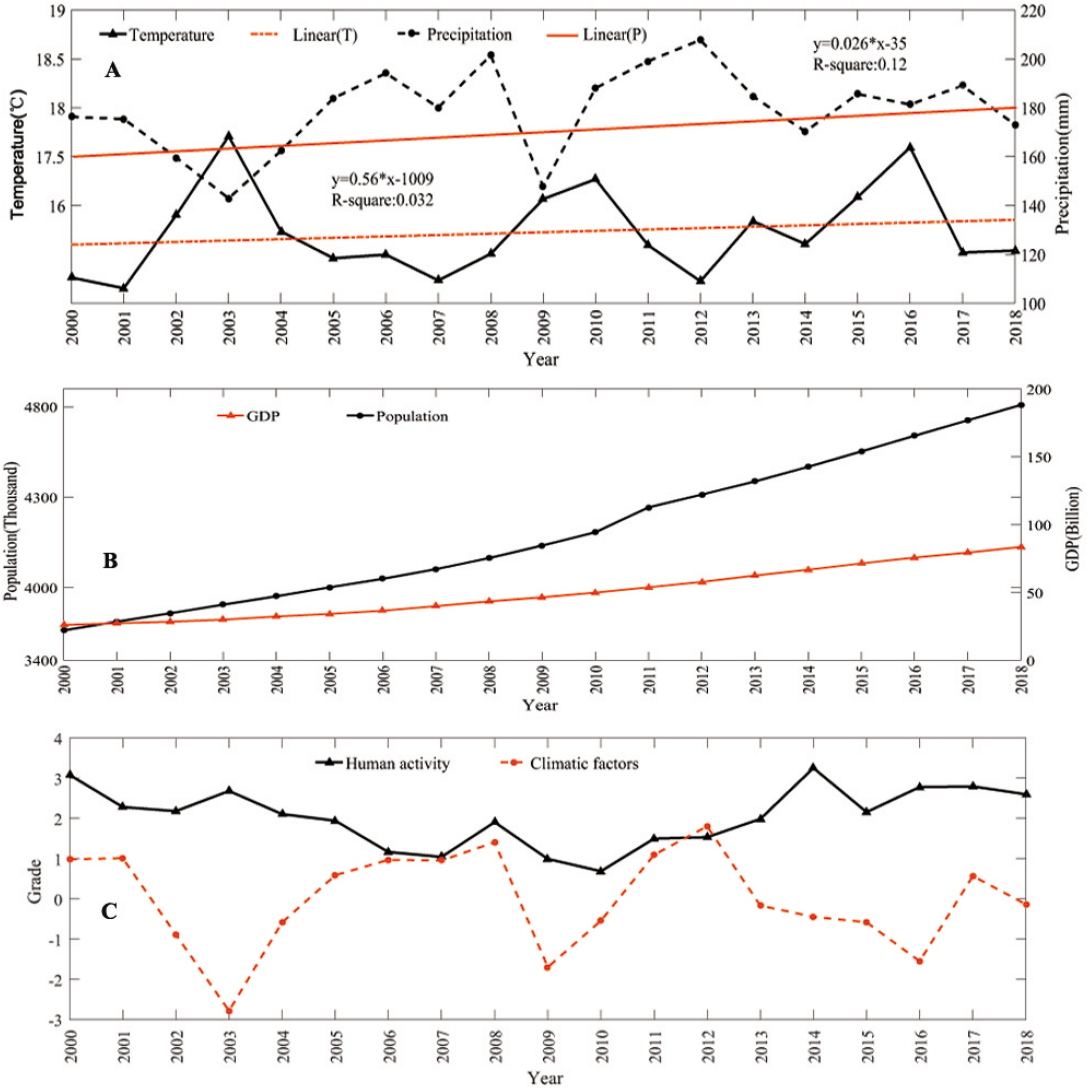
Driving forces of natural vegetation cover. (A) Changes in annual average temperature and total annual precipitation; (B) changes in total population and GDP; and (C) principal component scores of human activities and climate factors.

Climate change profoundly affects the growth and distribution of plants, temperature affects the growth cycle of vegetation, and precipitation affects the composition and physiological changes of vegetation. The Aral Sea is in a semi-arid area, and the climate has a greater impact on vegetation cover changes. As shown in Fig. 8A, the temperature growth rate from 2000 to 2018 is 0.56 °C/a, which indicates climate warming in this region in the past 19 years. However, the temperature in winter showed the opposite trend, and the temperature in the Pamirs dropped significantly. The region has a climate with hotter summers and colder winters. The precipitation in this area also has a slight increase trend, and there is a clear increasing trend of precipitation in Kazakhstan, where Rain Fed Crops account for 10.12% of the total cultivated land area in Central Asia (Lioubimtseva, 2014).

#### Impact of human activities on natural vegetation cover

The Aral Sea Basin is located in five Central Asian countries. Since the disintegration of the Soviet Union in 1991, the economic and social patterns of the region have varied. Among them, agriculture has changed from state-owned to private and free operations, and human activities inevitably affect the growth of regional vegetation. Population quantity, distribution, migration and human production activities can destroy the surface and land cover, and the physical and chemical properties of soil and water. They are closely related to changes in regional vegetation cover. The state of economic development reflects the degree of urbanization and land use in the region. In the past nineteen years, the GDP growth rate was 6.7% (Fig. 8B).

#### Driving forces of natural vegetation cover changes

We selected five factors to determine natural vegetation cover changes: annual average temperature, total precipitation, total population, GDP, and cultivated land area. PCA was used to quantitatively analyze the impact of various factors on vegetation coverage. The total population, GDP and arable land area contributed 0.82 to the first principal component, which can somewhat reflect the impact of human activities on vegetation coverage. The annual average temperature and annual total precipitation contributed 0.87 to the second principal component, which can somewhat represent the impact of climate factors on regional vegetation (Table 4). In the past nineteen years, the impact of climatic factors on the Aral Sea Basin generally initially increased and then decreased. The impact of human factors on the vegetation coverage of the Aral Sea Basin initially decreased and then increased (Fig. 8C). Overall, human activities were the major driving factor that affected the changes in vegetation cover in the Aral Sea Basin, of which the change in cultivated land area had a higher impact. Among the natural factors, precipitation was the main factor affecting the change in vegetation coverage in the Aral Sea Basin, followed by temperature.

**Table 4:** Principal component analysis results.

Ingredient	Eigenvalues	Contribution rate/%	Cumulative contribution rate/%
1	2.4876	0.4975	0.4975
2	1.4457	0.2891	0.7866
3	0.6816	0.1363	0.9229
4	0.3839	0.0769	0.9998
5	0.0012	0.0002	1.0000

Compared with natural factors, the impact of human activities on vegetation cover changes is more rapid and direct. According to statistics, the petrochemical, non-ferrous metallurgical, and power industries in the region show a growth trend, while the output value of the light and wood industries shows a downward trend. The rapid industrialization has caused substantial damage to vegetation coverage. Regional economic development has a profound impact on regional population growth and migration. Combining the results of the Mann–Kendall test and Sen median trend analysis can effectively characterize the trend changes of ground features. By analyzing the change curve of natural vegetation and arable land, we found that the area of arable land increased in 2004–2005, 2007–2013 and 2015–2018; the vegetation area decreased. Jiang et al. (2017) also pointed out that the expansion of irrigated farmland led to the degradation of some natural vegetation. The Aral Sea Basin is also a hot spot for abandoned farmland. The residents of the former prosperous ports migrated to Kazakhstan and Uzbekistan due to the dryness of the Aral Sea, and a large amount of arable land was abandoned. Abandoned farmland was gradually restored to other vegetation types.

### Accuracy verification

**Figure 9:**
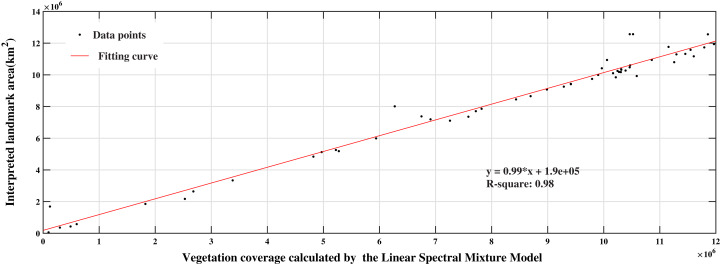
Two-dimensional scatter diagram of calculated and interpreted values.

To reflect the accuracy of the analysis results, we used the remote sensing data of Landsat 7 Collection 1 Tier 1 with a spatial resolution of 30 m. The auxiliary data is Google Earth images. The combination of the two images can distinguish the types of surface features in the study area and create a high-precision classifier (Yun et al., 2010; Löw et al., 2018; Pauleus & Aide, 2020). Based on the ArcGIS platform, we randomly selected 80 sampling points in the study area to establish a circular buffer with a radius of 1,000 m. We conducted visual interpretation of shape files based on Landsat 7 and Google Earth images, and calculated the pixel area of objects in the buffer area and compared them with the area calculated by the abundance map in the same buffer area. The vegetation coverage area calculated by LSMM was taken as the abscissa, and the interpretation result was taken as the ordinate. The scatter plot is shown in Fig. 9. The scatter points are evenly distributed near the line with a slope of 1.

The root mean square error (RMSE), determination coefficient (*R*^2^) and SSIM parameters were used to quantitatively evaluate the experimental results.

From Fig. 9, there are more points at the lower end of the ${y} = {x}$ line than at the upper end, indicating that the vegetation coverage calculated by the LSMM is higher than the vegetation coverage interpreted by Landsat. However, most of the points are distributed on the line *R*^2^ = 0.98, showing that the calculated value has a significant relationship with the interpreted value. RMSE is small, representing a slight difference between the calculated and interpreted values. The SSIM is 0.88, indicating that there was a significant similarity between the calculated planting area and the interpreted value.

## Discussion

### Vegetation dynamics

With the decrease in water, the change in vegetation coverage in the Aral Sea Basin is a matter of concern. Because the watershed is located at the junction of five countries, detailed information on vegetation coverage is difficult to obtain using traditional measurement methods (Lioubimtseva, 2014). In this study, by using a linear regression model to analyze the temporal and spatial changes in vegetation coverage, we found that the overall natural vegetation coverage in the Aral Sea Basin has continued to increase with the decrease in water bodies since 2000. This result indicates that the vegetation coverage in the Aral Sea area has improved, but Piao et al. (2011) and Jiang et al. (2017) showed that the vegetation in the Aral Sea area was degraded. There are two main reasons for the inconsistent results: (1) Previous studies were conducted at different scales, focusing on part of the Aral Sea basin or the entire Central Asian region (Xi & Sokolik, 2016; Jiang et al., 2017; Xu, Wang & Yang, 2017); (2) A variation in time spans. Some studies have shown that the Aral Sea Basin had an obvious degradation trend before 2000 (Crighton et al., 2013; Piao et al., 2011). We focused on 2000–2018. The vegetation improvement areas are mainly located in Kyrgyzstan, Tajikistan and along the riverbanks. Degraded areas are mainly located in southern Kazakhstan, where the oil, natural gas and chemical industries are thriving, but their extraction and transportation processes destroy the surface vegetation (Karnieli et al., 2008), especially sparse vegetation.

### Causes of vegetation dynamics

At present, the driving factors of vegetation change in this area are still unclear, and previous studies are limited to the impact of climate change on vegetation coverage (De Beurs et al., 2015). The study of vegetation cover does not omit the interference of cultivated land and artificial green space on the exploration of vegetation cover driving forces. The change in the coverage of cultivated land and artificial green space is determined by human activities. When discussing the driving force of vegetation cover change, cultivated land affects the analysis results. Therefore, distinguishing between cultivated land and natural vegetation is an important part of exploring the driving force of vegetation coverage. Climate and human activities jointly affect vegetation change, both of which need to be considered when exploring the driving forces of vegetation coverage. Climatic and human factor analysis concluded that the reason for the change in natural vegetation cover from 2000 to 2018 is the development of irrigated farmland in desert areas. This is clearer in northern Turkmenistan and southern Kazakhstan desert areas. Using Google Earth software, we found that there is abandoned farmland in Eastern Kazakhstan, Amu Darya, and lower reaches of the Sylar River, accounting for 13% of the irrigated farmland in the Aral Sea Basin, which is also suitable for farming. Studies have shown that approximately one-third of river water is diverted to cultivated land, and unreasonable irrigation of farmland is also a major reason for the decrease in water volume in the basin. In particular, the irrigation of farmland in Uzbekistan and Kazakhstan is a key factor affecting the decline of the Aral Sea (Löw et al., 2018).

Since the mid-1990s, the temperature in Central Asia has been rising rapidly and is currently the highest on record (Davi et al., 2015). Rising temperatures accelerate the melting of glaciers and replenish water sources. Kyrgyzstan has begun to implement afforestation policy, mainly including the Batken area, the Djalal-abad area, and the Naryn area, where the afforestation area is also increasing with an annual growth rate of 8%. These data indicate that national policy decisions and production inputs in the past twenty years may also affect vegetation coverage. Population dynamics, industrial structure, and other factors may also play an important role in vegetation dynamics, and further research on the spatial pattern of vegetation dynamics is needed. At the same time, abandoned arable land is gradually restored to vegetation (Hauck et al., 2016). The increased precipitation and glacial melt water cannot compensate for the water loss of the Aral Sea (Yang et al., 2020), and the increase in natural vegetation coverage and decrease in desert area do not mean that the ecological crisis in the region is alleviated. The environmental problems of the Aral Sea Basin need to be studied further.

### Comparative results

**Figure 10:**
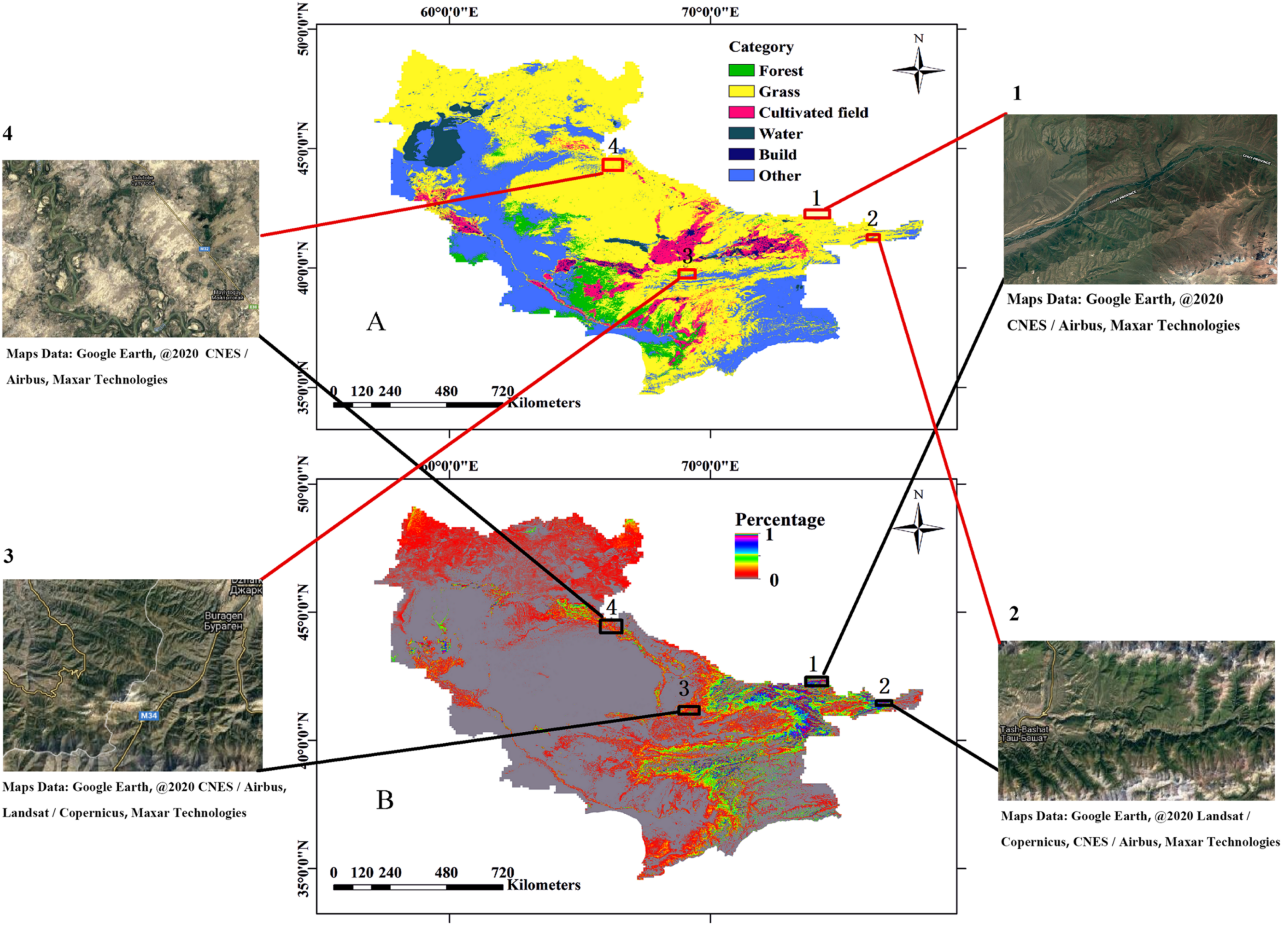
Comparative Results. (A) MCD12Q1 feature classification product data; (B) natural vegetation coverage calculated by LSMM. Images 1, 2, 3 and 4 are satellite image (Obtained from Google Earth) of corresponding positions, which are used for accuracy verification.

MCD12Q1 is a land cover type product of the MODIS tertiary data. The land cover dataset contains seventeen major land cover types, including vegetation and arable land (Zhang & Roy, 2017). However, during the application process, we found that the update time of the data product lags the original data of MODIS, and there is a phenomenon of inaccurate classification of individual features. We compared the natural vegetation cover abundance map calculated by LSMM with the product data of MCD12Q1. The areas with inconsistent classifications were verified using Google Earth data. As shown in Fig. 10, there are forests in the 1, 2, 3 and 4 point area, and the same area in the MCD12Q1 is shown as grass or other features. The results show that the natural vegetation cover abundance map calculated by the LSMM is reliable.

## Conclusions

Based on the remote sensing image data of the GEE platform and strong storage calculation ability, this study uses MOD13A1 as the data source and Landsat 7 Collection 1 data as the verification data. LSMM was used to calculate the vegetation coverage of the Aral Sea Basin and the analysis reached the following conclusions:

1. Correlation analysis of changes in the area covered by local objects showed that the increase in cultivated land area has a significant relationship with the decrease in desert area. The area where the spatial distribution of cultivated land area increases is similar to the area where the desert is significantly reduced. It also shows that the main reason for the decrease of downstream water is the draw of water to cultivated land.
2. With the reduction of water, the natural vegetation coverage in the Aral Sea Basin has shown an upward trend from 2000 to 2018, and the vegetation coverage increased by 17.77% during this period. The improvement areas of natural vegetation are mainly distributed in the eastern mountain areas (Tianshan and Pamir) and delta coasts.
3. Human activity has been a major factor in the vegetation cover changes in the Aral Sea Basin in the past nineteen years, and the migration of cultivated land is the dominant factor. Among the climatic factors, the main factor affecting the change in vegetation coverage in the Aral Sea Basin is precipitation, followed by temperature.

## Supplemental Information

10.7717/peerj.10747/supp-1 Supplemental Information 1 Population, GDP and cultivated land area data.

10.7717/peerj.10747/supp-2 Supplemental Information 2 Precipitation and temperature data.

10.7717/peerj.10747/supp-3 Supplemental Information 3 Code.

## Additional Information and Declarations

### Data Availability

The following information was supplied regarding data availability: The raw measurements and code are available in Supplemental Files.
